# Determination of Diethyl Phthalate and Polyhexamethylene Guanidine in Surrogate Alcohol from Russia

**DOI:** 10.1155/2011/704795

**Published:** 2011-05-17

**Authors:** Yulia B. Monakhova, Thomas Kuballa, Jenny Leitz, Dirk W. Lachenmeier

**Affiliations:** ^1^Chemisches und Veterinäruntersuchungsamt (CVUA) Karlsruhe, Weissenburger Stra*β*e 3, 76187 Karlsruhe, Germany; ^2^Department of Chemistry, Saratov State University, Astrakhanskaya Street 83, 410012 Saratov, Russia

## Abstract

Analytical methods based on spectroscopic techniques were developed and validated for the determination of diethyl phthalate (DEP) and polyhexamethylene guanidine (PHMG), which may occur in unrecorded alcohol. Analysis for PHMG was based on UV-VIS spectrophotometry after derivatization with Eosin Y and ^1^H NMR spectroscopy of the DMSO extract. Analysis of DEP was performed with direct UV-VIS and ^1^H NMR methods. Multivariate curve resolution and spectra computation methods were used to confirm the presence of PHMG and DEP in the investigated beverages. Of 22 analysed alcohol samples, two contained DEP or PHMG. ^1^H NMR analysis also revealed the presence of signals of hawthorn extract in three medicinal alcohols used as surrogate alcohol. The simple and cheap UV-VIS methods can be used for rapid screening of surrogate alcohol samples for impurities, while ^1^H NMR is recommended for specific confirmatory analysis if required.

## 1. Introduction

The consumption of surrogate alcohols (i.e., illegal alcohol not originally intended for human consumption) from different sources is a significant problem in Russia [1–3]. Several cases of poisoning by alcohol-containing liquids were recorded during the last decade [4]. Surrogate alcohols (disinfectants, medicinal alcohols, and perfumes) from unidentified sources were consumed in all cases [4, 5]. During chemical investigation of the consumed beverages, diethyl phthalate (DEP) and polyhexamethylene guanidine (PHMG) were found [5]. PHMG has been used as an effective antiseptic compound, and DEP has been applied as denaturing agent for ethyl alcohol (denaturing of alcohol is undertaken for the purposes of exemption from excise duty that is applied to nondenatured forms, see [1] for details). Therefore, these compounds may be contained in surrogate alcohols prepared from disinfectants or denatured alcohols. In this situation, particular attention should be paid to developing fast methods for quantitative and qualitative analysis of the two compounds in alcohol samples.

Qualitative analysis of DEP can be carried out based on the reactions with pyrogallol in acidic medium or with potassium hydroxide [4]. DEP can be further quantified in alcoholic beverages with gas chromatography (GC) with mass spectrometry (MS) [6] or flame-ionization detector (FID) [7]. These chromatographic methods are very sensitive and accurate but also time consuming and expensive. However, for the determination of typical amounts of DEP in beverages that contain denatured alcohol (200–1270 mg/L [6, 7]), the sensitivity of trace GC analysis is not required and it might be possible to develop screening methods based on spectroscopic measurements. However, a recent review [8] and our literature search indicate that direct spectroscopic techniques (such as ^1^H NMR and UV-VIS) have not been studied for phthalates determination so far.

There are very few methods for PHMG determination. Nessler's reagent can be used for qualitative analysis of PHMG [4]. Another method for detecting PHMG in aqueous solutions is VIS spectroscopy with Eosin Y as indicator [9]. The absorbance of the ionic derivative formed in the acidic medium is measured at 535 nm. The method has advantages of simple operation and high accuracy. The problem here is the lack of commercial availability of a pure PHMG standard for calibration purposes. 

Thus, the aim of this paper was to develop and evaluate analytical approaches for screening of DEP and PHMG in alcoholic beverages. In particular, the sample preparation and analytical procedure itself should be fast and inexpensive because the method should be usable in routine alcohol testing laboratories in Russia. This study will therefore evaluate two spectroscopic techniques—nuclear magnetic resonance (NMR) and UV-VIS absorbance spectroscopy—which are powerful tools for the simultaneous identification and quantification of compounds in complex mixtures [10–13]. The procedure was applied for the analysis of authentic beverages from the Russian Federation.

## 2. Experimental Section

### 2.1. Samples

A total of 22 samples were analyzed. Samples included commercial vodkas (*n* = 4), medicinal alcohol (*n* = 7), homemade samogon (*n* = 7), and surrogate alcohol (*n* = 4). All samples were offered for human consumption. The sampling itself was performed in three cities (Saratov, Lipetsk, and Irkutsk) in the Russian Federation. The sampling was conducted in the context of an international project designed to characterize the quality and toxicity of alcoholic beverages, especially unrecorded alcohols. Details on sampling and toxicological evaluation of the public health implications will be published elsewhere.

### 2.2. Chemicals and Reagents

Diethyl phthalate (DEP) was purchased from Merck (Darmstadt, Germany). A stock standard solution of DEP was prepared at a final concentration of 1.50 mg/mL (UV-VIS) or 50 mg/mL (^1^H NMR) in ethanol. Calibration solutions of DEP were prepared by diluting the standard solution in ethanol/water solution (6 : 4 and 7 : 3 for UV and ^1^H NMR measurements correspondingly).

### 2.3. UV-VIS Method

Solutions for qualitative UV-VIS screening were prepared by dilution of an aliquot of the alcohol beverage with 60% ethanol and were directly analysed. For the quantitative polyhexamethylene guanidine (PHMG) analysis, we applied a spectroscopic procedure with Eosin Y as an indicator based on the study by Chmilenko et al. [9]. The method takes advantage of the phenomenon that the guanidine group in the PHMG can change the color of tetrabromofluorescein (Eosin Y) from orange to pink. For analysis, 40 *μ*L of the alcoholic beverage is mixed with 5 mL of standard buffer solution (pH 4.0) (Merck, Darmstadt, Germany) and 40 *μ*L of 0.10% solution of Eosin Y (Sigma-Aldrich, Germany). Then the volume was adjusted to 10 mL with distilled water. All spectrophotometric measurements were performed on a Perkin Elmer Lambda 12 dual beam spectrometer equipped with an automatic cell changer. The spectrometer was operated with the UV WinLab software (version 2.80.03). The spectra were acquired in the range between 190 and 700 nm with a data interval of 1.0 nm. All measurements were made against ethanol (60% vol) as a blank.

### 2.4. ^1^H NMR Method

All ^1^H NMR measurements were performed on a Bruker Avance 400 Ultrashield spectrometer (Bruker Biospin, Rheinstetten, Germany) equipped with a 5-mm SEI probe with Z-gradient coils, using a Bruker Automatic Sample Changer (B-ACS 60). All spectra were acquired at 300.0K. An 8-fold suppression of all the signals of water and ethanol was conducted. The data were acquired automatically under the control of ICON-NMR (Bruker Biospin, Rheinstetten, Germany), requiring about 20 min per sample.

^1^H NMR spectra for nontargeted analysis were acquired using a Bruker noesy pulse sequence with 32 scans and 4 prior dummy scans. The sweep width was 20.5 ppm and the time domain of the FID was 65kB. For non-targeted analysis, 300 *μ*L of beverage is mixed with 50 *μ*L of ethanol, 190 *μ*L of distilled water, and 60 *μ*L of pH 7.4 NMR buffer containing 0.1% TSP as internal standard (Bruker Biospin, Rheinstetten, Germany). The mixture is then poured into an NMR tube and is directly measured. 

For targeted DEP quantification, 300 *μ*L of beverage is mixed with 60 *μ*L of NMR buffer, and then the volume is adjusted to 600 *μ*L with ethanol (70%). The mixture is then poured into an NMR tube and is directly measured using the same sequence as above. For quantification of DEP, two multiplets in the 7.76–7.65 ppm region were integrated and summarized. The samples were quantified using standard addition.

For confirmation of the presence of PHMG, the sample was evaporated to dryness using a rotary evaporator and the residue was dissolved in DMSO-d6 with TMS (0.1%) as internal standard (PHMG cannot be measured using ^1^H NMR in aqueous solution due to rapid proton exchange with the solvent protons). The DMSO spectra were recorded using 64 scans, 20.5 ppm sweep width, and 2 prior dummy scans.

### 2.5. Statistics

All ^1^H NMR spectra were phased and baseline-corrected prior the analysis. Different statistical packages were used. In particular, Unscrambler X version 10.0.1 (Camo Software AS, Oslo, Norway) was used for Multivariate Curve Resolution—Alternating Least Squares (MCR-ALS). MCR is a soft-modeling method that focuses on describing multicomponent mixtures without a priori information about the system [14]. Furthermore, a technique for multivariate curve resolution [12, 14] based on Independent Component Analysis (ICA) [15] was used. MCR-ALS and ICA are statistical tools which can be applied to multivariate data sets to extract information (both spectra and relative concentrations) of the most significant mixture components [14]. Some applications of ICA are reported in analytical chemistry for studying environmental samples or foods [10, 13]. The ICA algorithm used in this paper consists of taking the first derivative of each mixture spectrum and then computing the inverse of the mixture matrix for these derivative spectra using the MILCA method [16]. For the execution of the ICA calculations, we applied Matlab v. 7.0 (The Math Works, Natick, Mass, USA). To assess the similarities between the resolved and the experimental spectra, we used Pearson's correlation coefficient (R). Statistical significance was assumed at below the 0.05 probability level.

### 2.6. ^1^H NMR and UV Spectra Calculations

As no standard substance of PHMG was commercially available, spectra calculation methods have to be used for spectral assignment.

^1^H NMR spectra calculations were carried out using ChemBioDraw 12.0 software (CambridgeSoft, Cambridge, UK). Chemical shifts are estimated for all hydrogen atoms for which additivity rules are available. Following a hierarchical list, the algorithm first identifies key substructures of a molecule. A substructure provides the base value for the estimated shift. For details, see [17].

We used HyperChem Professional (Hypercube, Gainesville, FL, USA) software package (v.8.0) for quantum chemical calculations to predict UV-VIS spectra. The main goal of all quantum-chemical methods is solving of the Schrödinger equation. It is based on Hartree-Fok-Rutan equation by self-consistent field (SCF) method. We applied semiempirical PM3 (Parametrised Model 3) method for calculation with full geometry optimization. In most cases it is the most accurate semiempirical method. The main approaches of PM3 method include adiabatic, one-electron, MO LCAO (molecular orbital as a linear combination of atomic orbitals) and INDO (Intermediate Neglect of Differential Overlap) approximations. For UV-VIS spectra calculation we used 5 occupied and unoccupied orbitals in configuration interaction. For details regarding the calculations, see [18].

## 3. Results and Discussion

### 3.1. Qualitative UV-VIS Prescreening of Alcoholic Beverages

**Figure 1:**
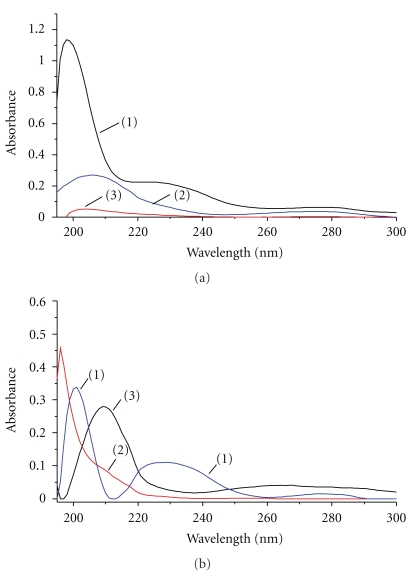
Absorbance spectra of surrogate alcohol (1) (1/150 dilution), samogon (2), and vodka (3) (dilution 1/10) (a); results of MCR application for spectra of alcoholic beverages: DEP (1), PHMG (2), and interference (3) (b).

As UV-VIS spectroscopy is today one of the cheapest, simplest, and most robust tools for chemical analysis of solutions, we first evaluated this method for pre-screening of our unrecorded beverages without any sample preparation besides dilution (Figure 1(a)). Samogon samples have higher absorbance in the UV region than vodkas which is characteristic of the presence of different compounds (e.g., higher alcohols, aromatic compounds) that are formed in the beverages during the fermentation process and that cannot be removed using the rudimentary distillation procedure applied during homeproduction [19]. Compared to the typical samogon and vodka samples, surrogate alcohols have additional spectral bands with the maximum absorbance around 195 and 225 nm and intensive absorbance in the region of *π*→π* transitions (260–280 nm). Typically medicinal alcohols have very strong absorbance in the UV spectral range because of the presence of herbal extracts (e.g., hawthorn, see below).

However, reliable assignments of spectral bands cannot be made because of the extensive overlap in the UV region. To cope with this problem, MCR-ALS was applied to a set of 11 beverages (2 vodkas, 5 samogon, and 4 surrogate alcohols) (Figure 1(b)). Statistical analysis revealed the presence of three pure components. One pure compound represents a complex spectrum where several bands are present (*λ*_max_ 195, 226, and 276 nm). We were able to identify this component as DEP (the correlation coefficient (R) between the experimental spectra of a pure standard and the resolved spectra is 0.98). The other resolved component has an absorbance only in the 190–210 nm range and was identified as PHMG. This assignment was made based on the relative MCR-ALS concentrations and the fact that only one investigated sample contains PHMG (as confirmed by derivatization and ^1^H NMR, see below). We also performed semiempirical quantum chemical calculation of the UV-VIS spectrum of PHMG for a further confirmation. It has been found that the maximum of the calculated spectral band would be 195 nm, which is very similar to the experimental value (190 nm). The third component in Figure 1 refers to other substances that can be found in homemade alcoholic beverages. 

On this stage we can conclude that UV spectroscopy can easily differentiate commercial vodka from surrogate alcohol and homemade samogons and allows to preselect samples for further quantitative investigation.

### 3.2. UV-VIS Derivatization and ^1^H NMR for Quantitative Guanidine Determination

During analysis of our samples with Eosin Y as indicator we confirmed the presence of PHMG in the one sample of surrogate alcohol identified above. Due to the fact that a commercial standard is not available and that the synthesis of PHMG requires special conditions (high temperature, vacuum atmosphere [20]) not feasible in our laboratory, we used an indirect spectro-chemometric method for PHMG quantification in the positive sample.

**Figure 2:**
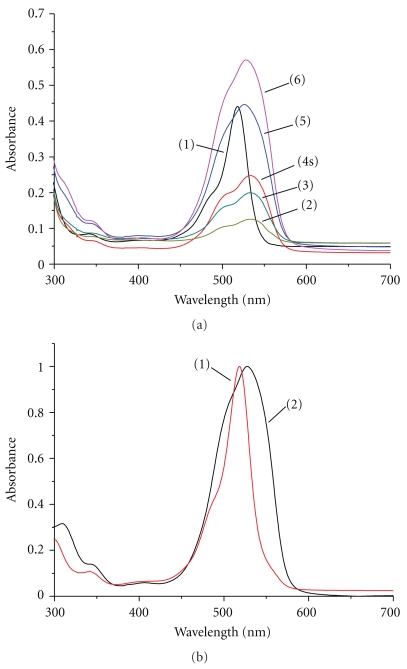
Experimental absorbance spectra of Eosin Y without (1) and with (2–6) the presence of surrogate alcohol containing PHMG; c (Eosin) in mg/L: 0.82 (2); 1.63 (3); 3.26 (1, 4); 6.52 (5); 8.16 (6) (a); Resolved MILCA spectra of Eosin Y (1) and PHMG—Eosin Y complex (2) (b).

Absorbance spectra of the Eosin Y-PHMG derivative (contained in the sample) solutions are presented in Figure 2(a). In the presence of PHMG, the band of Eosin (*λ*_max_ 517 nm) is broadened and shifted to the longer wavelengths (*λ*_max_ 525 nm). This is indicative of the fact that a polyelectrolyte associate (PHMG—Eosin Y) is formed [9]. The MILCA algorithm applied to the spectra of Eosin Y-PHMG solutions revealed the presence of two independent components—Eosin Y and its polyelectrolyte associate (PHMG—Eosin Y) in the mixtures (Figure 2(b)). The Pearson's correlation coefficient (R) between the experimental and resolved spectra of Eosin Y is 0.97. Based on the known total concentration of Eosin Y in the solutions and the (Eosin Y)/(PHMG-Eosin Y complex) concentration ratios (obtained during ICA analysis) we calculated the amount of PHMG in the surrogate alcohol to be 515 ± 50 mg/L (Table 1). Thus, application of multivariate techniques can simplify the analytical procedure of PHMG determination and makes it possible to determine PHMG in alcoholic beverages without a standard solution.

**Figure 3:**
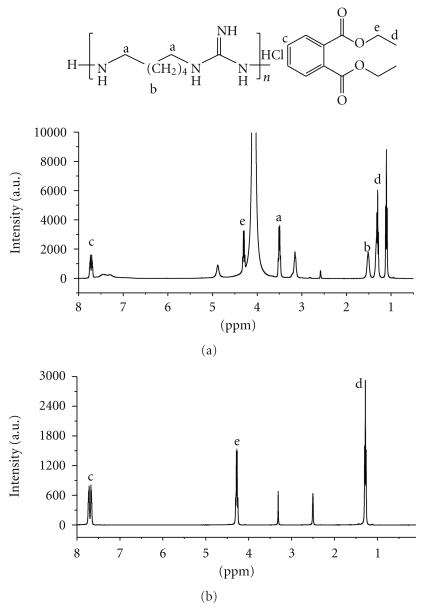
^1^H NMR spectra of DMSO extract of surrogate alcohol (a) and DEP standard (b).

For final confirmation of the presence of PHMG in the surrogate alcohol sample we used ^1^H NMR spectroscopy. ^1^H NMR spectra of the DMSO extract of the surrogate alcohol are shown in Figure 3. The spectra show characteristic signals of PHMG and DEP (the sample with PHMG also contains DEP). In particular, the signals at 3.25–3.05 ppm (methylene protons a), 1.61–1.22 ppm (methylene protons b) correspond to PHMG. The presence of DEP does not lead to interferences with PHMG integration as there is no overlap between signals (see Figure 3 and below). To confirm the assignment of the spectral bands, we calculated ^1^H NMR spectra of PHMG (because no pure standard substance is available). *δ*H_cal_ 1.29 ppm and 1.52 ppm confirmed the presence of methylene protons b, whereas *δ*H_cal_ 2.87 ppm indicate signals of methylene groups a (Figure 3). The spectra obtained also resemble PHMG spectra available in the literature [21].

It is finally possible to indirectly estimate the quantity of PHMG using the signals of DEP in the ^1^H NMR spectra as reference, because for DEP a pure standard is available. We used the integrals of aromatic and methyl proton signals for quantification. We found that the concentration of PHMG (496 mg/L) determined in this fashion is in good accordance with the one obtained with UV-VIS spectroscopy (Table 1).

### 3.3. ^1^H NMR and UV Quantification of DEP

In ^1^H NMR, signals from DEP occur at 1.00–1.20 ppm (methyl protons d), 4.32–4.22 ppm (methylene protons e), and 7.75–7.60 ppm (aromatic protons c). While signals in the aliphatic and mid-field ranges cannot be used for quantification of this compound (because of the strong overlap with the signals of other compounds present in the alcoholic beverages), signals in the aromatic range appear to be best for this purpose.

Table 2 summarizes the method validation results for DEP. The ^1^H NMR assay was linear in the concentration range of 100–7000 mg/L. When determined according to DIN 32645 [22], the limit of detection (LOD) was 90 mg/L and the limit of quantification (LOQ) was 181 mg/L. 

According to the ^1^H NMR results, only two of the 22 analyzed authentic alcoholic beverages from Russia contained DEP in concentrations of 1269 and 275 mg/L (Table 1). We also reconfirmed the absence of DEP in the 255 samples analyzed previously using GC/MS and confirmed it in the 2 positive samples from Lithuania [6]. The concentrations found in the current study were comparable with those found in Russian samples from Kyzyl [7] and Lithuanian unrecorded alcohols [6].

During UV-VIS prescreening of our samples it was shown that DEP can also be determined in the UV region (Figure 1). We developed and validated the method for the determination of DEP in alcoholic beverages in this region. The results for method validation and DEP quantification were presented in Tables 1–3. The ^1^H NMR spectroscopy showed relative standard deviations ranging between 5.3 (spiked sample at 500 mg/L, *n* = 5) and 7.1% (authentic sample at 1344 mg/L, *n* = 5). The precision of the UV-VIS method depends on the wavelength selected for quantification, and details are shown in Table 3. The validation results of both methods appear to be acceptable for the purpose. However, the UV-VIS assay has lower LOD and LOQ. Furthermore, UV-VIS spectroscopy is generally considered to be cheaper and faster than the ^1^H NMR procedure.

However, the lack of selectivity of the UV-VIS spectroscopy in respect to DEP analysis must be pointed out as disadvantage. As mentioned above, other compounds may absorb at the targeted wavelengths (226 and 276 nm) (Figure 1(a)). However, typically DEP is present in denatured alcohol in higher concentrations than all other compounds absorbing in this range (Table 1). Therefore the necessary dilution (1/100) before analysis compensates the lack of selectivity caused by spectral overlap, as it moves the absorbance of the interferences below the detection limit. Nevertheless, positive UV-VIS results should be confirmed using a more specific assay such as NMR or GC/MS. Next, it is interesting to compare our methods with previously published techniques. In general, quantitative methods for phthalates determination include sensitive techniques such as gas and liquid chromatography. All start with concentration prior to chromatographic analysis because phthalates are mostly found at trace levels (**μ**g/L or less). This step is considered as the most challenging part for phthalates analysis [8]. The proposed extraction and clean-up procedures are often very tedious and thus there is a higher chance for artefactual determination caused by contamination from phthalates found ubiquitously in materials and reagents. The levels of phthalates in denatured alcohol are considerably higher than those found in the other products [6–8]. Therefore, no preparation step besides a dilution is required. Thus, the developed spectroscopic methods that have a smaller number of steps minimize the probability of contamination.

### 3.4. Qualitative ^1^H NMR Analysis for Hawthorn Presence

**Figure 4:**
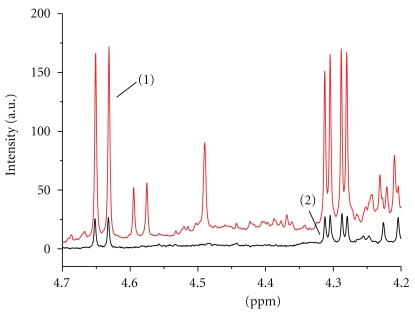
^1^H NMR spectrum in the 4.70–4.20 ppm range of medicinal alcohol (1) compared with hawthorn plant material (2).

Finally, all samples were screened for unknown substances using ^1^H NMR spectroscopy (so-called nontargeted analysis) [23]. In the ^1^H NMR spectra of the medicinal alcohols, several signals that could not be assigned were found. Hawthorn (*Crataegus spp.*) tincture is the most common form of medicinal alcohol used as surrogate alcohol on the Russian market [24, 25]. The medicinal alcohols in our sample were also labeled as “haw”. To confirm the presence of hawthorn, we measured ^1^H NMR spectra of hawthorn plant material (available from pharmacies as herbal tea) for comparison. We can conclude that signals at 2.05–2.00 ppm and 2.70–2.64 ppm (aliphatic range); 3.93–3.90 ppm; 4.35–4.32 ppm; 4.66–4.60 ppm and 5.24–5.22 ppm (mid-field range); 7.10–7.00 ppm (aromatic range) in three of the medicinal alcohol derive from the presence of substances from hawthorn extract. As an example, the spectra of a medicinal alcohol in comparison with hawthorn extract in the mid-field range are compared in Figure 4.

## 4. Conclusions

In this paper two new methods based on different types of spectroscopy (^1^H NMR and UV-VIS) for quantification of DEP and PHMG were developed. These methods were validated and introduced to determine DEP and PHMG contents in unrecorded alcohols from Russia. The methods were proven capable of providing robust results. Our validation shows that the relative measurement errors are generally less than 10%. Our results are indicative of the suitability of ^1^H NMR and UV-VIS spectroscopic techniques coupled with advanced chemometric methods (MCR-ALS, ICA) as a reliable analytical tool in routine analysis of alcoholic beverages. The nontargeted character of the ^1^H NMR method also allows to assign other compounds such as in the case of medicinal alcohols containing hawthorn.

Especially the UV-VIS method has the potential to improve consumer safety, as it allows to rapidly determine health-relevant compounds in alcohol samples. The toxicological aspects and public health implications of our findings of DEP and PHMG in alcohols consumed by humans will be evaluated in a future study.

## Figures and Tables

**Table 1:** Results of DEP and PHMG quantification in unrecorded alcohol samples from Russia (*n* = 5).

Sample	DEP (mg/L)		PHMG (mg/L)	
	UV-VIS	^1^H NMR	UV-VIS	^1^H NMR
Surrogate alcohol	1344 ± 85	1269 ± 90	515 ± 50	496
Medicinal alcohol	311 ± 24	275 ± 21	n.d.	n.d.

n.d.: not detectable (for limit of detection see Table 2).

**Table 2:** Comparison of method validation results for DEP.

Parameter	UV-VIS at 227 nm^a^	^1^H NMR
LOD (mg/L)	2.5	90
LOQ (mg/L)	10	181
Linear range (mg/L)	2-150	100–7000

^a^The sensitivity is approximately a factor of 10 higher at 227 nm than at 276 nm.

**Table 3:** Precision and accuracy of the UV/VIS method for analyzing DEP in alcohol samples.

Sample	Wavelength (nm)	RSD Intraday (%) (*n* = 5)^a^	RSD Interday (%) (*n* = 7)	Recovery (%) (*n* = 5)
Authentic surrogate alcohol sample from Russia (1344 mg/L)	227	1.8	2.5	—
	*276 *	*1.1*	*6.8*	—

Authentic medicinal sample from Russia (311 mg/L)	227	0.9	0.9	—
	276	4.5	4.6	—

Authentic surrogate alcohol sample from Lithuania (608 mg/L)	227	(interference)
	276	1.9	3.0	—

Spiked vodka sample (500 mg/L)	227	1.0	1.2	100
	276	5.5	6.6	105

Spiked vodka sample (1000 mg/L)	227	1.3	1.6	95
	276	2.3	2.8	105

^a^RSD: relative standard deviation.
